# Temperature Field Analysis for PZT Pyroelectric Cells for Thermal Energy Harvesting

**DOI:** 10.3390/s111110458

**Published:** 2011-11-02

**Authors:** Chun-Ching Hsiao, Jing-Chih Ciou, An-Shen Siao, Chi-Yuan Lee

**Affiliations:** 1 Department of Mechanical Design Engineering, National Formosa University, No. 64, Wunhua Rd., Huwei Township, Yunlin County 632, Taiwan; E-Mails: opportunity8246@gmail.com (J.-C.C.); qbasic147@gmail.com (A.-S.S.); 2 Department of Mechanical Engineering, Yuan Ze Fuel Cell Center, Yuan Ze University, No. 135, Yuan-Tung Rd., Taoyuan 320, Taiwan; E-Mail: cylee@saturn.yzu.edu.tw (C.-Y.L.)

**Keywords:** pyroelectricity, PZT, thermal energy, temperature variation rate

## Abstract

This paper proposes the idea of etching PZT to improve the temperature variation rate of a thicker PZT sheet in order to enhance the energy conversion efficiency when used as pyroelectric cells. A partially covered electrode was proven to display a higher output response than a fully covered electrode did. A mesh top electrode monitored the temperature variation rate and the electrode area. The mesh electrode width affected the distribution of the temperature variation rate in a thinner pyroelectric material. However, a pyroelectric cell with a thicker pyroelectric material was beneficial in generating electricity pyroelectrically. The PZT sheet was further etched to produce deeper cavities and a smaller electrode width to induce lateral temperature gradients on the sidewalls of cavities under homogeneous heat irradiation, enhancing the temperature variation rate.

## 1. Introduction

Energy conversion devices and harvesting systems, converting environmental energy sources such as light, vibration, wind power, thermal energy and mechanical waste energy into electrical energy, have been widely developed because micro-actuators and micro-sensors need an independent and embedded power source for large deployment of wireless network operations. Therefore, an efficient energy conversion cell has become a crucial requirement.

Thin-film pyroelectric devices have many advantages, such as easy integration with on-chip circuitry, un-cooled detection, room-temperature operation, high speed, low system cost, portability and wide spectral response with high sensitivity. They have been successfully used in many applications, such as pollution monitoring, hot image detector devices, intruder alarms and gas analysis [1–4]. However, pyroelectricity has often been abandoned as a practical energy source due to its low efficiency [5]. Recently, one study focused on pyroelectric generators based on fabricated screen-printed PZT and commercial PVDF films [6], which suggested interesting possibilities for their use in micro-power generators, producing up to 0.5 mJ of energy, enough to power typical autonomous sensor nodes. It is possible to improve the design of pyroelectric converters by increasing the thickness of the PZT cells, decreasing electrical capacitance of the PZT cells and using PZT cells poled with a higher electrical field during the fabrication process.

Moreover, achievement of an accurate thermodynamic cycle efficiency has also been studied in pyroelectric crystals [7]. A pyroelectric energy converter uses P(VDF-TrFFE) co-polymer and the Olsen cycle to directly convert waste heat into electricity [8], achieving a maximum energy density of 130 J/L at 0.061 Hz with temperature oscillations between 69.3 and 87.6 °C. Therefore, pyroelectric energy conversion offers a novel and direct way to convert waste heat into electricity by alternatively heating and cooling a pyroelectric material, thus producing electricity.

The principle of pyroelectric devices is based on the pyroelectric effect, converting temperature variation to a corresponding electrical signal. The dynamic pyroelectric response current of pyroelectric devices can be described as [1]:

(1) $$i_{p}=\eta \times P\times A\times \text{dT}/\text{dt}$$

where η is the absorption coefficient of radiation, P is the pyroelectric coefficient of the pyroelectric materials, A is the electrode area and dT/dt is the temperature variation rate of the pyroelectric materials. The thermal-isolation structure, sensitive material properties, top-electrode layout and absorption coefficient of pyroelectric devices are the most important performance-enhancing qualities.

From Equation (1), it can be seen that a higher temperature variation rate in pyroelectric films leads to a higher response current in the pyroelectric sensors. Moreover, a partially covered top electrode has been proven to result in a higher responsivity than a fully covered electrode because it allows the ZnO layer to be in direct contact with the heat source [9].

Using qualitative analysis, a web-type top electrode has been designed to enhance the responsivity of ZnO pyroelectric sensors [10]. A larger exposed ZnO layer area leads to more heat absorption and also to more dispersed top electrodes. Thus the design of the web-type top electrode can manage both the area of the exposed ZnO layer and the dispersion of top electrodes. The outer regions of the web-type electrode possess a larger exposed ZnO layer area while the inner regions possess a lower dispersion of top electrodes. Therefore, the temperature variation rate increases in the pyroelectric films when a partially covered top electrode is applied.

This concept has been further extended to the etching of a three-dimensional pattern on a responsive LiTaO_3_ element, with lateral temperature gradients induced on the sidewalls of the responsive element under homogeneous irradiation [11]. Thus, the temperature variation rate increases in the responsive element, which in turn increases the voltage responsivity of the pyroelectric sensors. Moreover, the temperature variation rate is difficult to extract from thin films by experimental measurement. A finite element model has been built from the commercial software ANSYS to explore the temperature variation rate in pyroelectric elements with various thermal properties and geometries [12].

In this paper, a finite element model built by commercial multiphysics software COMSOL MULTIPHYSICS^®^ 3.5 was used to explore the temperature variation rate in commercial PZT pyroelectric cells. The electrode layouts and cavities in the PZT material were designed and implemented to enhance the temperature variation rate. Then, improvement in temperature variation rate of the PZT pyroelectric cells was evaluated by simulation and experiment.

## 2. Materials and Methods

### 2.1. Finite Element Model and Simulation

A PZT pyroelectric cell with dimensions of 38 mm × 38 mm × 0.214 mm was used. The cell comprised a 0.2 mm thick PZT sheet sandwiched between a top and a bottom electrode. The electrodes were 0.007 mm thick silver film. PZT samples were provided by ELECERAM TECHNOLOGY Co. The thick PZT sheet was used to generate more electricity than that produced by a thin PZT film [6].

**Figure 1.**
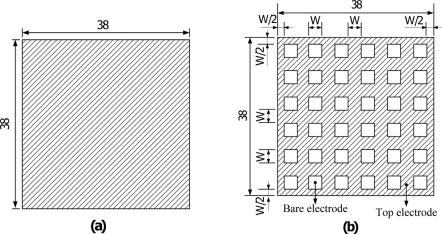
Top electrode dimensions in mm: (**a**) fully covered type; (**b**) mesh type.

**Figure 2.**
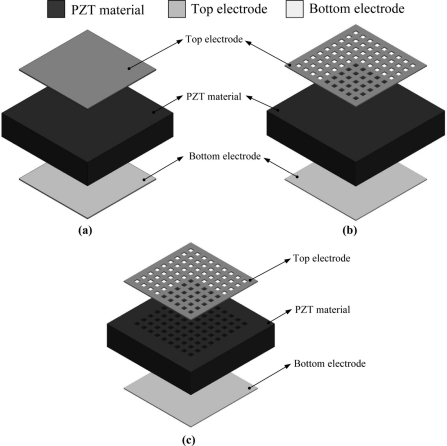
Schematic diagram of the PZT pyroelectric cells, (**a**) fully covered electrode, (**b**) mesh top electrode, (**c**) mesh top electrode with cavities.

The layout of the mesh top electrode had the same width ratio of the electrode and the bare electrode to monitor both the temperature variation rate and the top electrode area. The area of the mesh top electrode was about 1,083 mm^2^, while the electrode width size (W) was fixed at 50, 100, 200 and 500 μm, and the area of the fully covered top electrode was about 1,444 mm^2^. The dimensions of the top electrodes are detailed in Figure 1. Furthermore, the electrode width seemed to be related to thickness of the PZT sheet, which could then be optimized by exploring and analyzing the temperature variation rate in the PZT pyroelectric cell. Although the bare electrode was able to increase the heat absorption, etching and trenching the PZT under the bare top electrode zone were further enhanced to promote the temperature variation rate. Figure 2 shows a diagram of the PZT pyroelectric cells with various designs.

A two-dimensional finite element model was generated by the commercial multi-physics software COMSOL MULTIPHYSICS^®^ 3.5 to explore the temperature variation rate in a 200 μm thick PZT pyroelectric cell. The temperature variation rate is a dependent variable, and the electrode width (W) and etching depth (H) are given variables. The profile of cavities is assumed as an ellipse curve which is drawn by two sizes of the electrode width as the minor axis and the etching depth as the semi-major axis.

**Figure 3.**
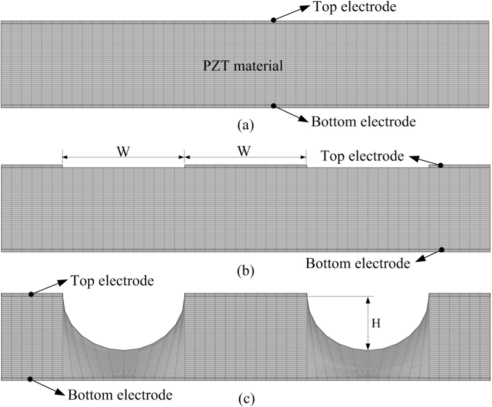
Finite element model for the PZT pyroelectric cells: (**a**) fully covered electrode, (**b**) mesh top electrode, and (**c**) mesh top electrode with cavities in the PZT sheet.

Material parameters of the PZT sheet and electrodes are listed in Table 1. All parameters were assumed to be isotropic. The models, which are shown in Figure 3, were meshed by regular mesh. The incident irradiation power applied on top side of the PZT pyroelectric cells was approximately 1.228 × 10^−12^ W/μm^2^ [12]. The thermal isolation condition was applied to backside of the PZT pyroelectriccells, and the symmetric condition was applied to the two lateral sides as boundary conditions.

### 2.2. Fabrication Process

**Figure 4.**
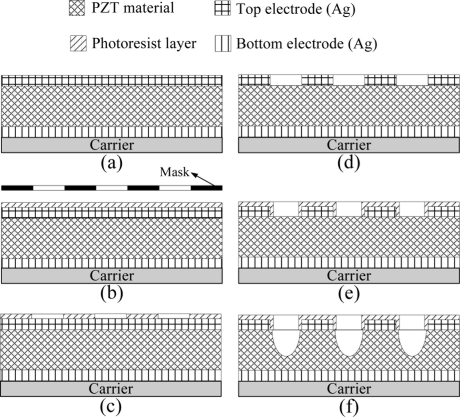
Process flow of the PZT pyroelectric cell: (**a**) a commercial PZT cell with dimension of 38 mm × 38 mm × 0.2 mm for pyroelectric generators attached to a glass substrate, (**b**) photoresist deposited on the top electrode by a spin coater and then exposed to UV light with a mask, (**c**) photoresist patterned by development, (**d**) top electrode etched with a shelter of the patterned photoresist layer using a wet etchant of HNO_3_: H_2_O = 7:3, (**e**) deposition and patterning of another photoresist layer with a smaller bare electrode width on the top electrode, and (**f**) PZT materials etched with a shelter of the patterned photoresist.

Properties the commercial PZT pyroelectric cell are tabulated in Table 2. Two strategies, patterning the top electrode and etching the PZT material, were used to promote the temperature variation rate of the PZT pyroelectric cells. Process flow of the PZT pyroelectric cells is shown in Figure 4. Firstly, the PZT pyroelectric cell was attached to a glass substrate, as shown in Figure 4(a). A photoresist manufactured by MICROPOSIT^®^ S1818 was deposited on the top electrode by a spin coater and then exposed to UV light with a mask, as shown in Figure 4(b). Patterning the photoresist was done by a developmental process, as shown in Figure 4(c). The top electrode was patterned using a shelter of the patterned photoresist layer and etched by a wet etchant with HNO_3_: H_2_O = 7:3, as shown in Figure 4(d).

**Figure 5.**
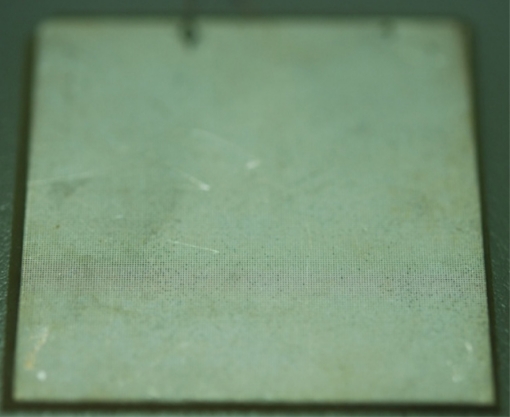
Fabricated pyroelectric cell.

Another photoresist layer with a smaller bare electrode width was then deposited and patterned on the top electrode, as shown in Figure 4(e). This process step was aimed to avoid the evacuation of PZT materials under the patterned top electrode, as an isotropy wet etching process was used to etch the PZT. Finally, PZT was etched using a shelter of the patterned photoresist layer to produce cavities in the PZT pyroelectric cells with a 0.5:5:10:50 mixture of HF-HCl-NH_4_Cl-H_2_O, as shown in Figure 4(f). The ammonium chloride (NH_4_Cl) was added to the etchant to reduce undercutting effects on the structure patterns [13]. Two samples (tabulated in Table 3) were used to verify the simulation results. Sample 1 was a commercial PZT pyroelectric cell with the fully covered top electrode, while the fabricated pyroelectric cell with an electrode width of 100 μm and an etching depth of 15 μm was named Sample 2 and is shown in Figure 5.

### 2.3. Measurement

**Figure 6.**
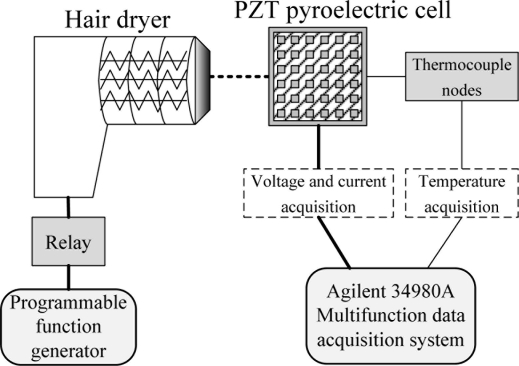
Schematic diagram of the measurement experiment.

A measurement system as shown in Figure 6 was used to evaluate the performance of the present PZT cells as pyroelectric generators. The thermal source was a hair dryer which was controlled by a relay and programmable function generator to produce time-dependent temperature variations (dT/dt). The hair dry worked as either a heater or as a fan. The PZT pyroelectric cell was held along the edges on a fixture in order to expose it completely to air. The distance between the hair dryer and PZT pyroelectric cells was about 20 mm. A type K (Chromel/Alumel) thermocouple was adopted to measure the temperature in the PZT pyroelectric cells, which was attached to the top electrode ensuring a good thermal contact. The output data of temperature, current and voltage were measured simultaneously by a computer-controlled data acquisition system (Agilent 34980A).

## 3. Results and Discussion

When a PZT pyroelectric cell is subjected to temperature variation, its internal polarization produces an electrical field which induces voltage or current response between the top and bottom electrodes. The response is proportional to the temperature variation rate in the PZT materials. A hair dryer used as a heat source must be chopped at a modulated frequency by a relay and programmable function generator to obtain a temperature variation rate in PZT pyroelectric cells.

**Figure 7.**
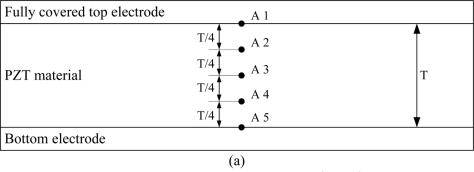

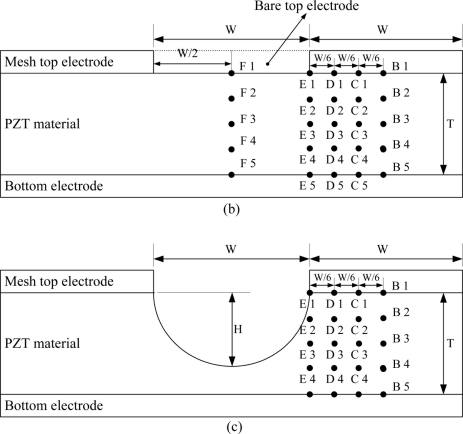
Points defined in the PZT materials: (**a**) fully covered electrode, (**b**) mesh top electrode, and (**c**) mesh top electrode with H-depth cavities.

Transient temperature fields in the PZT pyroelectric cells are simulated. The points shown in Figure 7 were used to explain the temperature variation rate in the PZT pyroelectric cells with the mesh top electrode, as compared to the fully covered top electrode. A1, B1, C1, D1, E1 and F1 were defined at the top of the PZT material. A3, B3, C3, D3, E3 and F3 were defined at the middle of the PZT material. A5, B5, C5, D5, E5 and F5 were defined at the bottom of the PZT material.

**Figure 8.**
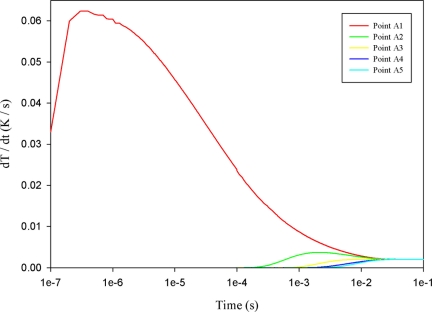
Simulated relationship between temperature variation rate (dT/dt) and time in the PZT material along the thickness of a fully covered electrode by.

Figure 8 shows the relationship between temperature variation rate and time in the PZT material at points A1 to A5, with a fully covered top electrode. When the point approached the top side of the PZT material, the temperature variation rate increased gradually, and its maximum peak moved leftward, thereby reducing the response time. Hence, point A5 had the lowest temperature variation rate in the PZT material using with fully covered top electrode.

**Figure 9.**
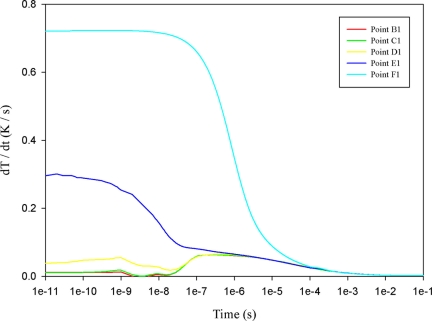
Simulated relationship between temperature variation rate (dT/dt) and time in the PZT material at points B1 to F1 for a mesh top electrode with a 100 μm electrode width.

**Figure 10.**
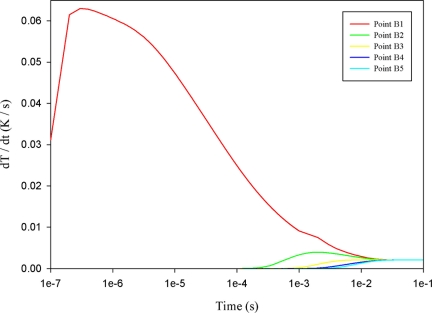
Simulated relationship between temperature variation rate (dT/dt) and time in the PZT material at points B1 to B5 for a mesh top electrode with a 100 μm electrode width.

When a mesh top electrode with a 100 μm electrode width was used to fabricate the PZT pyroelectric cell, point F1 showed direct contact with the irradiation source. The temperature variation rate at point F1 was obviously higher than that at points A1, B1, C1, D1 or E1, as shown in Figure 9. Moreover, the temperature variation rate at point E1 was higher than those at points B1, C1 and D1, because point E1 approached the uncovered top electrode directly to contact the heat source. Figure 10 shows the relationship between temperature variation rate and time in the PZT sheet at B1 to B5, with the temperature variation rate increasing gradually, with maximum peak moving leftward when the point approached the top side of the PZT sheet. Hence, point B5 had the lowest temperature variation rate in the PZT material with mesh top electrode. Moreover, the relationship between temperature variation rate and time in the PZT sheet from points B5, C5, D5 to E5 had no obvious change because the 200 μm thick PZT material possessed a larger thermal capacity and thus was disadvantageous to the temperature variation rate. However, a thicker PZT material proved helpful in enhancing the output response of pyroelectric converters [6]; hence, increasing the temperature variation rate at points B5 to E5 certainly enhanced the energy conversion efficiency of the PZT pyroelectric cells.

**Figure 11.**
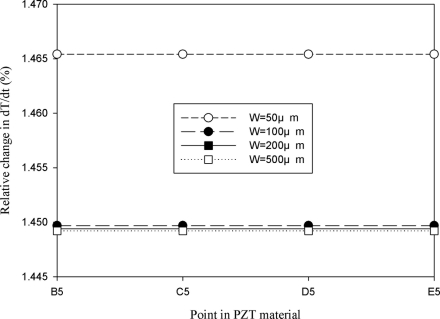
Simulated relative change in the maximum peak value of the temperature variation rate at points B5, C5, D5 and E5 for the mesh top electrode, as compared to that at point A5 for the fully covered electrode with various electrode widths.

Figure 11 shows the relative change in the maximum peak value of the temperature variation rate at points B5, C5, D5 and E5 for the mesh top electrode as compared to that at point A5 for the fully covered electrode under various electrode widths. The relative change in dT/dt can be expressed as:

(2) $$\text{dT}/\text{dt}(\%)=[{\left(\text{dT}/\text{dt}\right)}_{m}-{\left(\text{dT}/\text{dt}\right)}_{f}]/{\left(\text{dT}/\text{dt}\right)}_{f}\times 100\%$$

where (dT/dt)_m_ is the maximum peak value of the temperature variation rate at points B5, C5, D5 and E5 for the mesh top electrode, and (dT/dt)_f_ is the maximum peak value of the temperature variation rate at point A5 for the fully covered electrode. It showed a slight difference of approximately 1.4% at points B5, C5, D5 and E5 for the mesh top electrode, as compared to that at point A5 for the fully covered electrode with electrode widths of 50, 100, 200 and 500 μm. Therefore, altering the electrode width of a partially covered electrode to increase the heat absorption of PZT materials seems to only marginally improve the temperature variation rate of a thicker pyroelectric material.

**Figure 12.**
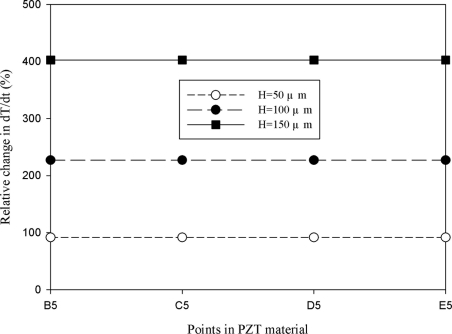
Simulated relative change in the maximum peak value of the temperature variation rate at points B5, C5, D5 and E5 for the mesh top electrode with cavities in the PZT material, compared with that at point A5 for the fully covered electrode, with an electrode width of 50 μm and etching depths of 50, 100 and 150 μm.

**Figure 13.**
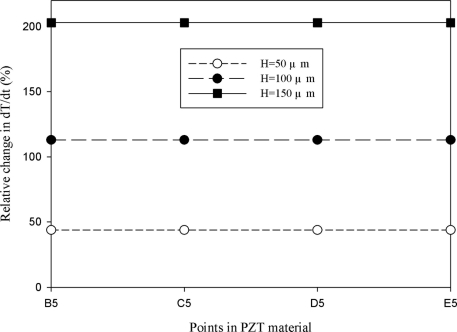
Simulated relative change in the maximum peak value of the temperature variation rate at points B5, C5, D5 and E5 for the mesh top electrode with cavities in the PZT material, compared with that at point A5 for the fully covered electrode, with an electrode width of 100 μm and etching depths of 50, 100 and 150 μm.

**Figure 14.**
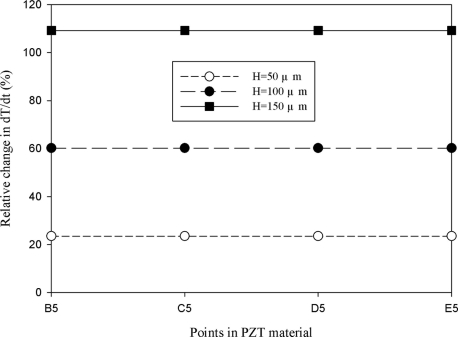
Simulated relative change in the maximum peak value of the temperature variation rate at points B5, C5, D5 and E5 for the mesh top electrode with cavities in the PZT material, compared with that at point A5 for the fully covered electrode, with an electrode width of 200 μm and etching depths of 50, 100 and 150 μm.

**Figure 15.**
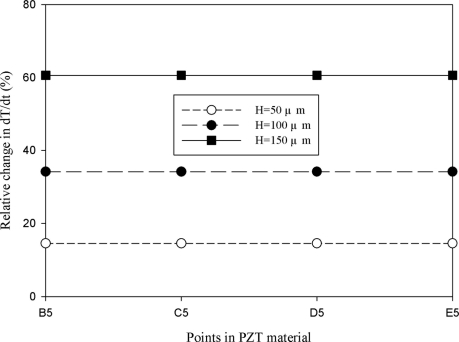
Simulated relative change in the maximum peak value of the temperature variation rate at points B5, C5, D5 and E5 for the mesh top electrode with cavities in the PZT material, compared with that at point A5 for the fully covered electrode, with an electrode width of 500 μm and various etching depths of 50, 100 and 150 μm.

A novel design to produce cavities in the PZT materials under the bare top electrode was adopted to enhance the temperature variation rate. Figures 12–15 show the relative changes in the maximum peak value of the temperature variation rate at points B5, C5, D5 and E5 for the mesh top electrode, compared to that at point A5 for the fully covered electrode with electrode widths of 50, 100, 200 and 500 μm and etching depths of 50, 100 and 150 μm. A deeper cavity and a smaller electrode width can effectively enhance the temperature variation rate in the PZT sheet because lateral temperature gradients were induced on the sidewalls of cavities under homogeneous heat irradiation. Moreover, the cavities produced more surface area and thereby increased heat absorption.

**Figure 16.**
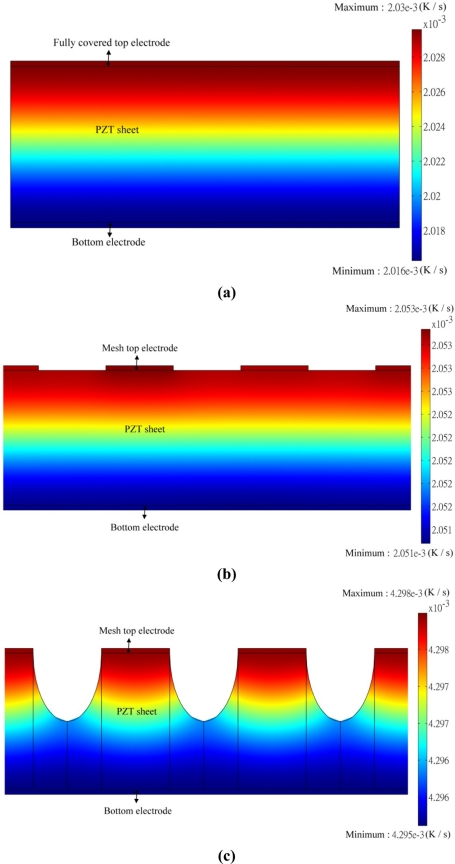
Simulated transient temperature variation field in the PZT pyroelectric cells: (**a**) fully covered electrode, (**b**) mesh top electrode, (**c**) mesh top electrode with cavities in the PZT sheet.

Figure 16(a) shows the transient temperature variation field at the maximum peak’s time of point A5 when the fully covered top electrode was used to fabricate the PZT pyroelectric cell. The temperature variation rate in the PZT sheet increased gradually toward the top electrode due to the incident radiation power applied on the top electrode. Figure 16(b) shows the transient temperature variation field at the maximum peak’s time of point B5 when the mesh top electrode with an electrode width of 100 μm was used to fabricate the PZT pyroelectric cell. The temperature variation rate in the PZT sheet with the mesh top electrode had no conspicuous increase compared to that of the fully covered top electrode, which was attributed to the 200 μm thick PZT material possessed a larger thermal capacity. Figure 16(c) shows the transient temperature variation field at the maximum peak’s time of point B5 when the PZT sheet possessed the mesh top electrode with an electrode width of 100 μm and the cavities with an etching depth of 100 μm was used to fabricate the PZT pyroelectric cell. The temperature variation rate in the PZT sheet with the mesh top electrode and the cavities had obvious increase about 110% compared to that with the fully covered top electrode or the mesh top electrode. The mesh top electrode collocated with the cavities in the PZT sheet was effective in enhancing the temperature variation rate in pyroelectric cells due to lateral temperature gradients on the sidewalls of cavities. Therefore, etching the PZT materials to produce cavities indeed improved the temperature variation rate.

An experiment was used to verify the simulation results. Table 4 summarizes the generated charge (Q), the generated charge per unit area (P_s_) and the maximum measured current (I_max_) for the pyroelectric cells with the fully covered electrode (Sample 1), compared to the mesh electrode with cavities in the PZT sheet (Sample 2). The charge was inferred from the integration of the positive area enclosed under the I curves when the temperature rose from 43 °C to 63 °C. P_s_ can be defined as the magnitude of the electrical polarization vector [6]. The measured electrical output of Sample 2 had a slight increase on Q and I_max_, compared to Sample 1 due to the fact that Sample 2 possessed the shallow cavities of 15 μm, even though the top electrode area of Sample 2 was less than that of Sample 1 by about 25%. Although the PZT etchant had a lower etching rate about 0.07 μm/min and was unsuitable for digging deeper cavities in the PZT sheet, the mesh electrode collocated with the cavities in the PZT sheet had a positive effect in improving the electrical output of the pyroelectric cells. Therefore, a manufacturing method to dig deeper cavities with a smaller electrode width in a thicker PZT sheet would be an interesting topic for further work.

## 4. Conclusions

A partially covered top electrode remarkably improved the responsivity of pyroelectric devices because the bare top electrode permits the heat source to be in direct contact with the PZT material. Altering the electrode width of a partially covered top electrode marginally improves the temperature variation rate in a thicker PZT material. However, a PZT pyroelectric cell with a thicker PZT material is beneficial in generating electricity pyroelectrically. Etching the PZT material to produce deeper cavities and a smaller electrode width can effectively enhance the temperature variation rate in a thicker PZT material, which can then improve the energy conversion efficiency of the PZT pyroelectric cells.

## Figures and Tables

**Table 1.** Material parameters used for finite element analysis.

**Material**	**Thermal conductivity (Wm**^−**1**^**K**^−**1**^**)**	**Specific heat (J g**^−**1**^**K**^−**1**^**)**	**Density (g cm**^−**3**^**)**	**Thickness (μm)**
**Top and bottom electrodes**	429	0.235	10.53	7
**PZT**	2.1	0.36	7.97	200

**Table 2.** Properties of the commercial PZT pyroelectric cells.

**Sample ID**	**Thickness (μm)**	**Area (mm^2^)**	**Size (mm × mm)**	**Relative dielectric constant (ε**_33_^T^/**ε**_O_)	**Density (g/cm^3^)**	**Poling field (V/μm)**
KA	200	1,444	38 × 38	2,100	7.9	3.5

**Table 3.** Samples for the PZT pyroelectric cells.

**PZT sample**	**Electrode type**	**Size (mm × mm)**	**Electrode width (W)**	**Etching depth (H)**
1	Fully covered electrode	38 × 38	none	none
2	Mesh electrode with cavities	38 × 38	100 μm	15 μm

**Table 4.** Experimental output for the PZT pyroelectric cells.

**PZT sample**	**Q (μC)**	**P_s_ (10**^−**2**^**C/m^2^)**	**I_max_ (μA)**
1 (Fully covered electrode)	23.8	1.64	3.28
2 (Mesh electrode with cavities)	25.1	2.31	3.51
